# Mobile-based Assessment of Entrustable Professional Activities in Urology Training: Implementation and Outcomes

**DOI:** 10.1016/j.euros.2025.01.013

**Published:** 2025-02-20

**Authors:** Fabian J. Aschwanden, Luca Afferi, Lukas Kandler, Adrian P. Marty, Philipp Baumeister, Agostino Mattei, Marko Kozomara, Christian D. Fankhauser

**Affiliations:** a University of Lucerne Lucerne Switzerland; b University of Zurich Zurich Switzerland; c University Hospital Balgrist Zurich Switzerland

**Keywords:** Medical education, Competency-based training, Entrustable professional activities, Mobile applications

## Abstract

**Background and objective:**

Urology residency programs often lack consistent feedback mechanisms and tracking of an individual’s progress. Competency-based medical education, using entrustable professional activities (EPAs), offers a solution but faces challenges in implementation. The aim of this study was to implement and assess a smartphone application for real-time EPA assessments in urology residency programs.

**Methods:**

A mobile application was introduced to 13 urology residents and ten supervisors at a Swiss training institution. Assessment characteristics were analyzed using descriptive statistics. Agreement between trainees and supervisors on task complexity and supervision levels was evaluated using Cohen’s and Fleiss’ κ metrics.

**Key findings and limitations:**

Over a period of 7 mo, 246 EPA assessments were recorded, of which 214 assessments were completed, representing a completion rate of 86%. Procedural EPAs accounted for 92% of the assessments, and nonprocedural EPAs for 8%. Cohen’s κ indicated moderate agreement for task complexity (κ = 0.56) and supervision levels (κ = 0.55). Higher agreement was observed when trainees were rated competent in supervising others (κ = 0.71). Limitations include the focus on procedural tasks and the small sample size.

**Conclusions and clinical implications:**

A mobile application can effectively facilitate real-time EPA assessments in urology training, promoting frequent feedback conversations and efficient tracking of resident progress. However, greater emphasis on nonprocedural EPAs is needed.

**Patient summary:**

We tested a mobile phone app that provides real-time feedback to urology residents and found that it enhanced their training experience. While the app effectively tracks progress in carrying out procedures, more focus is needed on developing nonprocedural skills such as patient counseling.

## Take Home Message

Mobile applications have the potential to facilitate effective real-time assessment of entrustable professional activities in urology training, with promotion of frequent feedback conversations and efficient tracking of a resident’s progress.

## 1 Introduction

Competency-based medical education is transforming residency training programs by shifting the focus from time- and quantity-based progression to the demonstration of competence in defined clinical activities. This approach addresses a critical gap in many residency programs, which often lack formalized, consistent feedback mechanisms. Such deficiencies make it challenging to ensure that each resident progresses towards independent practice in a timely manner. The limitations of traditional models are evident in countries such as Switzerland, where training in surgical specialties requires an average of 7.4 yr to complete, with nearly 20% of residents failing to achieve the necessary skills within the standard timeframe [1]. These statistics underscore the need for a more flexible, competency-driven approach to medical education that can adapt to individual learning rates and ensure timely acquisition of essential skills.

Entrustable professional activities (EPAs) have been introduced as a key component of competency-based medical education and provide a framework for assessing discrete, observable units of professional practice [2], [3]. Various methods for assessing EPAs in clinical settings exist, including supervisor evaluations, direct observations, and simulation-based assessments [4]. However, these approaches often face challenges such as delayed feedback, inconsistent evaluation criteria, and difficulty in tracking a trainee’s progress over time [5], [6]. In response to these issues, mobile technology has been used as a promising solution to enhance the efficiency, consistency, and immediacy of EPA assessments [7], [8], [9], [10].

We implemented use of a smartphone app to track EPA-based assessments in our urology residency program for real-time evaluation of learners using an entrustment-supervision scale. This scale has greater validity than traditional numerical scales [11], [12]. Here we describe the development and implementation of this mobile solution to key challenges in EPA assessment, as well as initial outcomes. To the best of our knowledge, this represents the first application of a mobile EPA assessment tool in urology residency, which is a pioneering step in competency-based medical education.

## 2 Materials and methods

The implementation consisted of two phases: (1) definition and development of the urology-specific EPAs; and (2) clinical implementation.

### 2.1 Phase 1: definition and development of urology-specific EPAs

A consultant working group at our institution defined 46 urology-specific EPAs. These EPAs cover core aspects of urology across various domains, including general tasks, specialty-specific tasks, preoperative, intraoperative, and postoperative phases, emergency situations, and nonoperative technical tasks.

The EPAs can be broadly categorized into procedural EPAs, which involve specific interventions (eg, transurethral resection of bladder tumor [TURBT]), and nonprocedural EPAs, which encompass skills such as patient counseling and other “soft” skills. The Supplementary material contains a full list of the EPAs.

### 2.2 Phase 2: clinical implementation

For phase 2, we integrated the preparedEPA smartphone app (PrecisionED, Wollerau, Switzerland) into our training program. The app, which is available on both the Google Play and Apple App stores, requires user registration. It was independently installed by 13 trainees and ten supervisors, encompassing all medical staff in our urology residency program, on their personal smartphones. While use of the app for EPA assessments was part of the clinical training routine, participation in anonymized data-sharing for research purposes remained voluntary.

The assessment process begins when a trainee has completed an EPA under supervision. The trainee opens the application, starts a new assessment, and selects the EPA performed from the predefined list. The app then generates a QR code for the supervisor to scan, which initiates the evaluation process. Both parties independently assess the complexity of the task and the level of supervision required for the trainee in future similar scenarios, each using their smartphone. To ensure simplicity and clarity for daily use, the level of supervision is rated on a five-point entrustment-supervision scale, ranging from observation (where the trainee is only allowed to observe the task) to supervise others (where the trainee is proficient enough to oversee other trainees performing the task; Table 1). After both parties have completed their ratings, the results are displayed on both smartphones side by side to show any agreement or disagreement. In addition, a training goal can be set if needed.

**Table 1:** Explanation of the different levels of supervision [2]

Level of supervision	Explanation
Supervise others	The trainee is allowed to supervise more junior learners
Distant supervision	The trainee is allowed to work unsupervised
Indirect supervision	The trainee is allowed to carry out an EPA without a supervisor in the room, but a supervisor is quickly available if needed (indirect reactive supervision)
Direct supervision	The trainee is allowed to execute an EPA with direct proactive supervision by a supervisor present in the room
Observe	The trainee is allowed to be present and observe but not perform an EPA

EPA = entrustable professional activity.

To continuously track progress, the assessment data (including task complexity and level of supervision for each EPA) are automatically aggregated and presented as a personalized, color-coded competency profile in the trainee’s portfolio. The competency profile can be shared with supervisors at the trainee’s discretion. Trainees maintain full control over who can access their profile and for how long they wish to share it.

For the scientific evaluation, we collected individual user data via the data extraction feature of the app. These data included the type of EPA, system time stamps, task complexity ratings, level of supervision ratings, and the decision to set a learning goal.

**Fig. 1:**
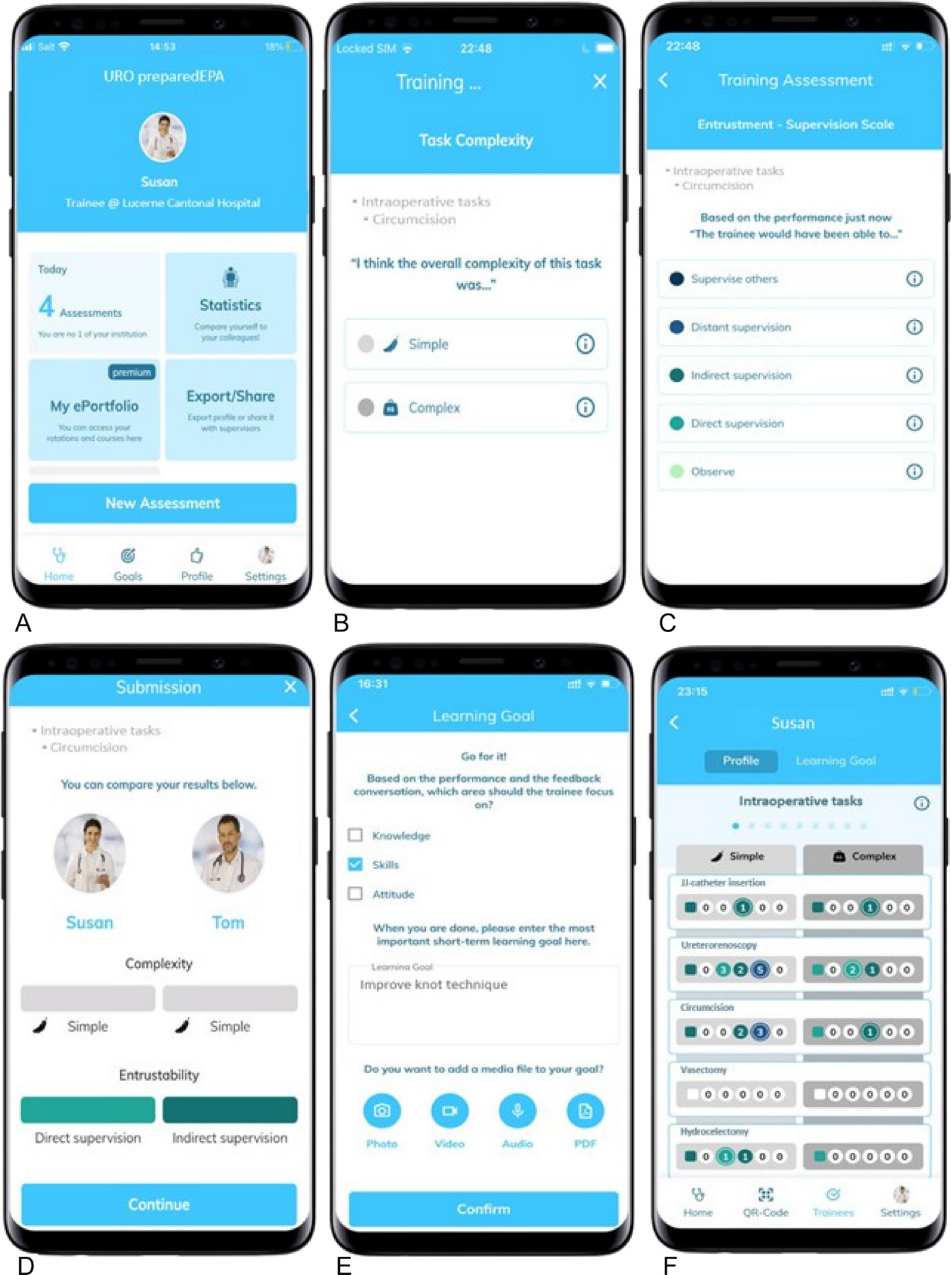
User interface of the mobile application. (A) The “New assessment” button initiates a new evaluation. (B) After selecting the specific entrusted professional activity from the predefined list (provided in the Supplementary material) and scanning the QR code generated (not shown in the figure), both the trainee and the supervisor must define the complexity of the task (simple vs complex). (C) In the next step, both the trainee and the supervisor select the level of entrustment on the supervision scale for future similar scenarios (observe, direct supervision, indirect supervision, distant supervision, supervise others). (D) The results are then displayed on the smartphones, indicating agreement or disagreement between the trainee and the supervisor ratings. (E) In addition, a specific learning goal can be set. (F) The competency profile, which can be shared with supervisors, gives an overview of the trainee’s competence for the different tasks.

A detailed visualization of the user interface and the workflow for assess an EPA is presented in Fig. 1.

### 2.3 Statistical analysis

Descriptive statistics were used to report assessment characteristics. To evaluate the agreement between trainees and supervisors, Cohen’s weighted κ was calculated. The strength of agreement was interpreted using the following scale for κ: ≤ 0 = agreement; 0.01–0.20 = slight agreement; 0.21–0.40 = fair agreement; 0.41–0.60 = moderate agreement; 0.61–0.80 = substantial agreement; and 0.81–1.00 = almost perfect agreement [13]. In an additional analysis, Fleiss’ κ was calculated as a measure of the agreement among the five levels of the entrustment scale: supervise others, distant supervision, indirect supervision, direct supervision, and observation. All statistical analyses were conducted using JASP v0.18.3.0 (JASP Team, Amsterdam, Netherlands).

## 3 Results

During the observation period from August 2023 until March 2024, we recorded 246 assessments for 13 trainees and ten supervisors. Of these, 214 assessments were completed, corresponding to a completion rate of 86%. Thirty-four (7%) were self-assessments and 212 (93%) were supervised assessments.

The median number of assessments was 18 (range 4–40) per trainee and 16 (range 4–64) per supervisor. The median completion time for an EPA assessment was 20 s (interquartile range 12–36) for trainees and 16 s (interquartile range 3–15) for supervisors. Learning goals were set in 18 cases (14%). Among the 46 EPAs available, procedural tasks were performed in the vast majority of cases (92%, *n* = 226), with nonprocedural EPAs rarely assessed (8%, *n* = 20).

The EPA most commonly assessed was ureterorenoscopy (23%, *n* = 57) followed by TURBT (13%, *n* = 33) and transurethral resection of the prostate (TURP; 10%, *n* = 25; Table 2). Trainees (64%) and supervisors (68%) mainly rated the complexity of the EPA performed as simple. Regarding agreement for task complexity, weighted Cohen’s κ values revealed moderate agreement between the trainees and supervisors(κ = 0.56, 95% confidence interval [CI] 0.47–0.69). There was also moderate agreement for the level of supervision (Cohen’s κ = 0.55, 95% CI 0.42–0.69). However, agreement differed substantially among the five categories for the level of supervision. There was substantial agreement on whether a trainee was entitled to supervise others (Fleiss’ κ = 0.712, 95% CI 0.57–0.85; Table 3). In general, trainee self-underestimation (23%) was more frequent than self-overestimation (13%).

**Table 2:** Assessment results

Parameter	Result
Number of assessments, *n* (%)	
Total number of assessments	246 (100)
Complete assessments	214 (86)
Type of assessment, *n* (%)	
Nonprocedural assessments	20 (8)
Procedural assessments	226 (92)
Ureterorenoscopy	57 (23)
Transurethral resection of bladder tumor	33 (13)
Transurethral resection of the prostate	25 (10)
Number of assessments per participant (*n*)	
Supervisors	10
Trainees	13
Median number of assessments per participant, *n* (range)	
Supervisors	16 (4–64)
Trainees	18 (4–40)
Median completion time per assessment, s (interquartile range)	
Supervisors	16 (3–15)
Trainees	20 (12–36)

IQR = interquartile range.

**Table 3:** Agreement between trainees and supervisors according to Fleiss’ κ by level of supervision

Level of supervision	Fleiss’ κ (95% CI)
Overall	0.49 (0.405–0.575)
Supervise others	0.712 (0.574–0.85)
Distant supervision	0.501 (0.363–0.639)
Indirect supervision	0.285 (0.147–0.423)
Direct supervision	0.586 (0.448–0.724)
Observation	0.393 (0.255–0.531)

CI = confidence interval.

## 4 Discussion

Our study suggests that use of a mobile application for competency-based medical education in urology training programs could revolutionize training and introduce a shift towards a competency-based learning environment. Efficiency features of the application include a median completion time of <1 min, which allows integration of feedback into daily clinical practice without imposing significant time burdens on trainees and supervisors. Previous research has suggested that trainees benefit more from frequent, brief feedback sessions rather than irregular, lengthy ones [14], highlighting the potential benefit of mobile applications.

A notable finding is the primary focus on procedural EPAs (92%) in comparison to nonprocedural EPAs (8%). This imbalance highlights the current focus of trainees on the number of procedures carried out (eg, TURP) and neglect of nonprocedural skills such as patient counseling, which are equally crucial to a physician’s competence [15]. Encouraging more frequent assessment of these competencies may lead to a more balanced evaluation of trainees' overall readiness for independent practice.

The moderate agreement between trainees and supervisors in evaluating the level of supervision suggests general alignment but also reveals areas of discrepancy. Interestingly, agreement was highest when indicating a trainee’s competence for supervision of others, possibly because this level represents a clear, observable milestone. The differences in the level of supervision ratings can serve as a basis for discussion and may motivate trainees to set learning goals [11].

An important benefit of this system is its potential to enhance the planning of surgical training and shift schedules. By providing multiple assessments of trainees’ competence by different supervisors in different clinical situations, the application allows planners to make informed decisions about which procedures a resident can safely perform independently, and which might require supervision or assistance. These rich and meaningful data allow more efficient and safer allocation of resources and responsibilities, ensuring that residents are appropriately challenged while maintaining patient safety.

However, the underutilization of the goal-setting feature, with learning goals set in only 14% of assessments, represents an area for improvement. Similarly, the median of 17 EPAs completed per trainee over 7 mo, while seemingly modest, represents a fourfold increase in comparison to the current annual minimum requirement for workplace-based assessments in Switzerland. This frequency is higher than in previous studies, indicating a positive trend for assessment frequency [9], [11]. Given the formative nature of competency-based medical education, encouraging more frequent use of this feature could enhance the educational value of EPA assessments and provide trainees with more specific guidance for improvement. This is the aim of further implementation science projects. The tendency towards self-underestimation by trainees contrasts with previous studies in other specialties, for which overestimation was more common [16]. This difference may be because of institutional, cultural, or methodological factors. While self-underestimation may be less risky for patient care than overestimation, it underscores the importance of accurate self-assessment and external validation via supervisory feedback.

On a broader scale, adoption of standardized EPAs could greatly enhance the efficiency of trainee recruitment and integration, particularly given the substantial variations in urology training both nationally and internationally [17], [18], [19]. Standardization would facilitate faster integration of new trainees into clinical roles by establishing clear expectations and competency requirements.

Limitations of our study include the small sample size, which may hinder the generalizability of the findings, and the focus on mainly procedural EPAs, which may not fully reflect the broad range of competencies required in urology. In addition, the nature of the app may have led to selection bias, with more technologically comfortable participants being more likely to complete assessments. Moreover, we did not analyze the cost effectiveness of the application and did not compare it to other applications available. Future research should address these limitations in larger studies with longer observation periods, a wider range of competencies, structured evaluation of user-friendliness and general perception of the app, and assessment of the cost/benefit ratio. Furthermore, an essential step will be to address ethical and privacy issues given the continuous tracking of trainees’ skills and knowledge via this technology.

In conclusion, our study demonstrated that use of an app for competency-based medical education has promise as a robust and efficient way for implementing EPAs in urology training programs. This approach facilitates more frequent and meaningful feedback, with high rates of task completion and rapid assessment times. However, challenges remain in achieving higher adherence and ensuring comprehensive assessment of both procedural and nonprocedural skills. Future studies should explore effective methods for integrating the app into daily clinical routine and expanding its use to cover the full spectrum of urological competencies.

## 5 Conclusions

Use of an app for competency-based medical education is a robust and efficient way to facilitate implementation of EPA in urology training programs. The application triggers more frequent and meaningful feedback, with high rates of task completion and rapid assessment times. However, challenges remain, particularly in achieving higher adherence and in ensuring that nonprocedural skills are assessed as rigorously as procedural tasks are. Future implementation studies should explore effective methods for integrating use of the app into daily clinical routines.

  ***Author contributions***: Christian D. Fankhauser had full access to all the data in the study and takes responsibility for the integrity of the data and the accuracy of the data analysis.

  *Study concept and design*: Fankhauser, Aschwanden, Kozomara.

*Acquisition of data*: Aschwanden, Kandler, Marty, Afferi.

*Analysis and interpretation of data*: Aschwanden, Fankhauser, Kozomara

*Drafting of the manuscript*: Aschwanden, Fankhauser, Baumeister.

*Critical revision of the manuscript for important intellectual content*: Fankhauser, Marty, Kandler, Afferi, Baumeister, Mattei, Kozomara.

*Statistical analysis*: Aschwanden, Fankhauser.

*Obtaining funding*: Fankhauser.

*Administrative, technical, or material support*: Mattei, Fankhauser, Kandler, Marty.

*Supervision*: Fankhauser, Mattei, Kozomara, Baumeister.

*Other*: None.

  ***Financial disclosures:*** Christian D. Fankhauser certifies that all conflicts of interest, including specific financial interests and relationships and affiliations relevant to the subject matter or materials discussed in the manuscript (eg, employment/affiliation, grants or funding, consultancies, honoraria, stock ownership or options, expert testimony, royalties, or patents filed, received, or pending), are the following: Adrian P. Marty and Lukas Kandler serve on the board of directors at PrecisionED Ltd, the company developing the preparedEPA application. The remaining authors have nothing to disclose.

  ***Funding/Support and role of the sponsor*:** This work was supported by a 2023 Gilead SAKK Expanding Horizons in Oncology Award to Christian D. Fankhauser. The sponsor played a role in preparation of the manuscript.
