# Supplementary material for: Mobile-based Assessment of Entrustable Professional Activities in Urology Training: Implementation and Outcomes
Source: Eur Urol Open Sci. 2025 Feb 20;73:71–6. doi: 10.1016/j.euros.2025.01.013 (PMC11889345; doi:10.1016/j.euros.2025.01.013)
Supplement: Supplementary Data 1 [file mmc1.docx]

**Supplementary material: EPA list**

General tasks

- Patient handover
- Ward round
- Patient counseling
- Multidisciplinary tumor board case presentation
- End of life situation

Urology specific tasks

- Diagnosis and treatment of voiding dysfunctions
- Diagnosis and treatment of erectile dysfunction
- Understanding of the pathophysiology of urolithiasis
- Prostate cancer screening
- Diagnosis and treatment of male infertility
- Urology consult

Preoperative tasks

- Patient admission
- Obtaining consent

Intraoperative tasks

- JJ-catheter insertion
- Ureterorenoscopy
- Circumcision
- Vasectomy
- Hydrocelectomy
- TURP
- TURB
- Urethrotomy
- Robot-assisted prostatectomy (table-side surgeon)
- Nephrostomy
- Cystoscopy
- Access for laparoscopic nephrectomy
- Abscess incision
- Frenuloplasty
- Negative wound pressure therapy management
- Prostate biopsy
- Access for robotic prostatectomy
- Access for robotic kidney surgery
- Median laparotomy
- Chevron incision
- Specimen retrieval / wound closure after laparoscopic / robotic intervention
- Abdominal fascia closure
- Inguinal orchiectomy

Postoperative task

- Postoperative management
- Patient discharge

Emergency situations

- Diagnosis and management of urosepsis
- Diagnosis and management of acute flank pain
- Diagnosis and management of scrotal pain

Non-operative technical tasks

- Insertion of a Foley catheter
- Insertion of a suprapubic catheter
- Sonography
- Fluoroscopy
- Urodynamic study
